# Exposure from the Chernobyl accident had adverse effects on erythrocytes, leukocytes, and, platelets in children in the Narodichesky region, Ukraine: A 6-year follow-up study

**DOI:** 10.1186/1476-069X-7-21

**Published:** 2008-05-30

**Authors:** Eugenia Stepanova, Wilfried Karmaus, Marina Naboka, Vitaliy Vdovenko, Tim Mousseau, Viacheslav M Shestopalov, John Vena, Erik Svendsen, Dwight Underhill, Harris Pastides

**Affiliations:** 1 Scientific Center for Radiation Medicine, Academy of Medical Sciences of Ukraine, Kyiv, Ukraine; 2 Department of Epidemiology and Biostatistics, Norman J. Arnold School of Public Health, University of South Carolina, Columbia, South Carolina, USA; 3 Radioecological Center, Ukrainian National Academy of Sciences, Kyiv, Ukraine; 4 College of Arts and Sciences, University of South Carolina, Columbia, South Carolina, USA; 5 Department of Environmental Health Science, Norman J. Arnold School of Public Health, University of South Carolina, Columbia, South Carolina, USA

## Abstract

**Background:**

After the Chernobyl nuclear accident on April 26, 1986, all children in the contaminated territory of the Narodichesky region, Zhitomir Oblast, Ukraine, were obliged to participate in a yearly medical examination. We present the results from these examinations for the years 1993 to 1998. Since the hematopoietic system is an important target, we investigated the association between residential soil density of ^137^Caesium (^137^Cs) and hemoglobin concentration, and erythrocyte, platelet, and leukocyte counts in 1,251 children, using 4,989 repeated measurements taken from 1993 to 1998.

**Methods:**

Soil contamination measurements from 38 settlements were used as exposures. Blood counts were conducted using the same auto-analyzer in all investigations for all years. We used linear mixed models to compensate for the repeated measurements of each child over the six year period. We estimated the adjusted means for all markers, controlling for potential confounders.

**Results:**

Data show a statistically significant reduction in red and white blood cell counts, platelet counts and hemoglobin with increasing residential ^137^Cs soil contamination. Over the six-year observation period, hematologic markers did improve. In children with the higher exposure who were born before the accident, this improvement was more pronounced for platelet counts, and less for red blood cells and hemoglobin. There was no exposure×time interaction for white blood cell counts and not in 702 children who were born after the accident. The initial exposure gradient persisted in this sub-sample of children.

**Conclusion:**

The study is the first longitudinal analysis from a large cohort of children after the Chernobyl accident. The findings suggest persistent adverse hematological effects associated with residential ^137^Cs exposure.

## Background

An explosion at the Chernobyl nuclear power plant on April 26, 1986, the worst accident in the history of nuclear power, resulted in radioactive pollution of much of the surrounding area. In the Ukraine, 2,293 villages and towns with a population of 2.6 million inhabitants were contaminated. A plume of radioactive fallout drifted over parts of Europe and reaching eastern North America, as well. Ever since, the public in these areas has been exposed to radiation, both externally and internally via contaminated locally-grown food, water and air.

Estimates of detrimental health effects from chronic radiation exposure vary widely [1]. Nearly 20 years after the Chernobyl disaster the World Health Organization in a report of the UN Chernobyl Forum found no evidence for an increased incidence of leukemia [2]. However, the same report found a complete lack of analytical studies in which dose and risks were estimated on an individual level. There were a few studies that analyzed white blood cells but most were based on a small number of children, focused mainly on micronuclei, and were often inconclusive [3-8]. Lenskaia *et al*., analyzed blood smears from 820 children living in a the Bryansk area in Russia and 46 controls from non-contaminated areas [9]. Using cytochemical assays (mucopolysaccharids) and esterase in 464 children with various exposure levels and 46 children from non-contaminated areas the work showed a reduction of mature T-lymphocytes and an increase of immature B-lymphocytes. In 1994–1996, Vykhovanets *et al*. and Chernyshov *et al*. studied T-lymphocytes in healthy children, 219 and 120, respectively, and children suffering from recurrent respiratory diseases (RRD) who resided around Chernobyl. Both studies compared the exposed groups with 148 non-exposed children, who were healthy or suffered from RRD. No information of leukocyte counts was provided [10,11].

Regarding red blood cells, we identified six published studies [5,12-16]. Stepanova *et al. *found more transitory, prehemolytic and degenerative forms of erythrocytes (red blood cells) in exposed children in comparison with control children [14]. Cross-sectional results on blood indices for years 1986, 1992 and 1998 were provided by Bebeshko *et al. *[16]. The authors examined children in the following age-groups: up to 3, 4–7 and 8–15 years old, residing in the Kiev, Zhytomyr, and Chernohiv provinces with ^137^Cs soil contamination density of 37 kBq/m^2 ^or less (37 kilo Bequerel/m^2 ^= 1 Curie (Ci)/km^2^) and contamination densities between 38 and 55 kBq/m^2^. The erythrocyte and leukocyte counts were significantly decreased in children aged 0–3 years living in Zhytomyr and in children age 8–15 years living in Chernihiv. The authors found no differences in hemoglobin, erythrocyte, leukocyte and platelet count in children residing in settlements with ^137^Cs soil contamination density of 38–555 kBq/m^2 ^compared to 37 kBq/m^2^.

The Chernobyl Sasakawa Health and Medical Cooperation project conducted the largest cross-sectional investigation of health effects in children [17]. Among a variety of markers, hematological outcomes were determined in 118,773 children residing in more than 80 regions with different radiation exposures from 1991 to 1996, including 779 children from the Narodichi region [18,19]. Each region represented a distinct exposed population rather than a regional sample of the larger exposed population. Children from each region were examined at a different points in time and there was no follow-up of each regional cohort in subsequent years. Some children were re-examined, but only those who presented a critical clinical outcome. The Chernobyl Sasakawa Health and Medical Cooperation project was a successful and comprehensive screening project. However, the project was not sufficiently designed to assess differences in health effects in regions and over time. For instance, the result of the exams in different regions and times, though presented as time trends, were confounded by different regions examined in different years [19]. In addition, improvements detected in the re-examinations of children were judged as recovery. This ignores the fact that exclusive examination of observations above a critical value will by chance produce 'recovery' due to chance movements of observations below the cut-off point in the re-exam (regression to the mean). Although the results of this study have never been reported in peer-reviewed scientific journals, the project has been influential when assessing the risk of the accident.

No study has yet investigated hematological follow-up data in children exposed from the Chernobyl accident. This motivated us to asses the association between residential soil density of ^137^Caesium (^137^Cs) and hemoglobin concentration, and erythrocyte, platelet, and leukocyte counts in repeated measurements taken from 1993 to 1998.

## Methods

### Population

**Figure 1:**
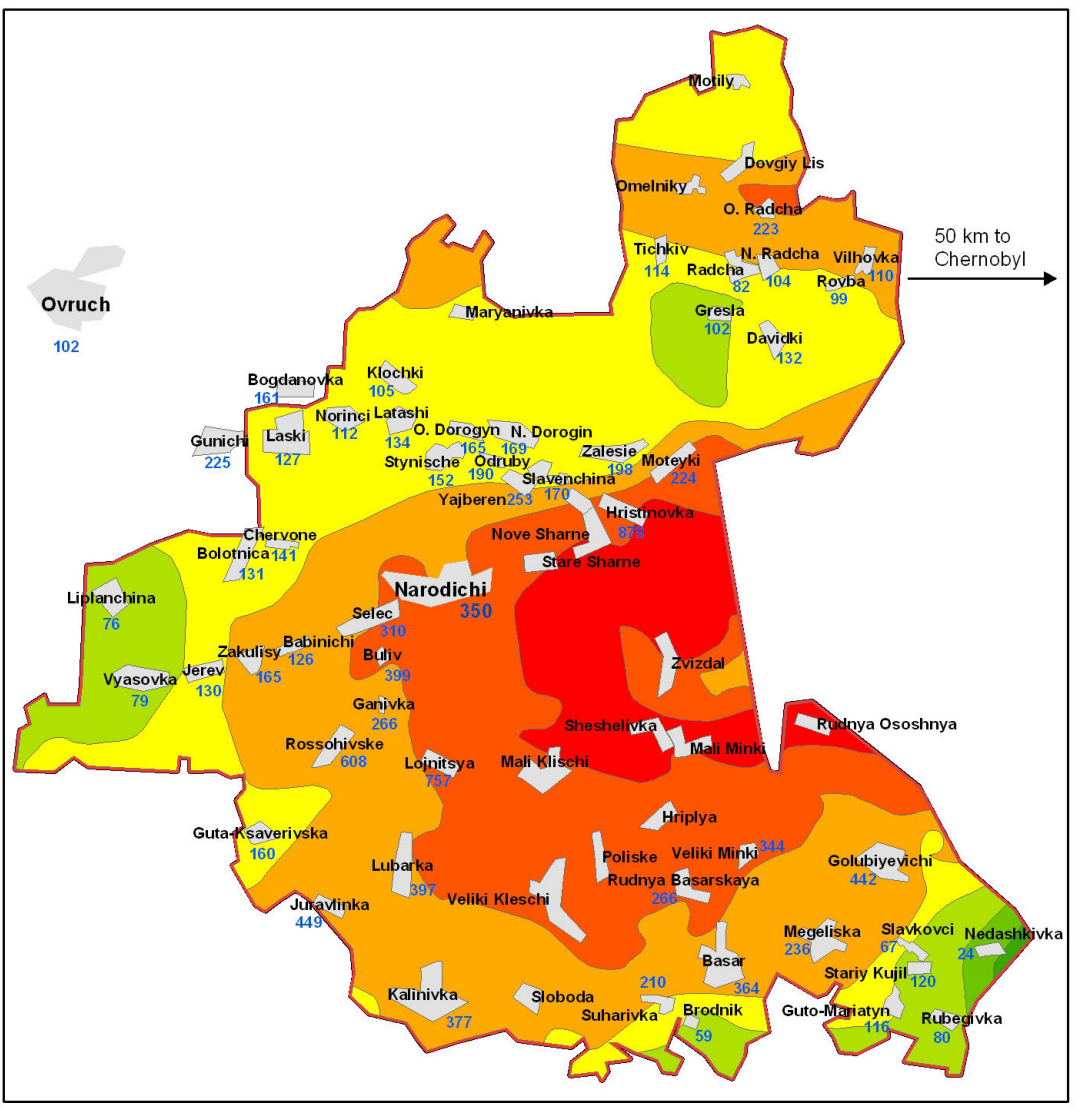
Map of the Narodychi region's territory soil pollution by ^137^Cs.

The settlements in the Narodichesky region (Zhitomir Oblast, Ukraine) are approximately 80 km from the Chernobyl nuclear site (Figure 1). Approximately 11,400 people reside in this region, including about 2,000 children [20]. Three quarters of its population live in rural villages, the others in small towns. The food supply is predominantly locally grown. To monitor the health effects of the accident, since 1986 every child from birth to age 18 in the contaminated territory was required to have a yearly medical examination. The Human Subject Committee at the University of South Carolina has approved these analyses.

### Exposure measurement

Following the Chernobyl accident, soil became contaminated with ^137^Cs, a nuclear fission product that has a half-life of 30 years, and emits both beta- and gamma-irradiation (Figure 1). The highest levels of ^137^Cs were found in the surface layers of the soil where it is absorbed by plants and mushrooms, thereby entering the local food supply. Dosimetric assessments of settlements in the Ukraine contaminated by radionuclides were published from 1991 to 2005 in 10 reports [21]. In addition, the Ministry of Emergency Situations provided spatial data on radiation [22]. Soil measurements used in this work represent the average of numerous measurements in each village over several years, in particular 1991–1993. These averages were officially named 'certifications' and documented in publications by the Ministry of Health. [21]. Since residential and individual ^137^Cs levels are highly correlated, we used the contamination in the residential area as an approximation of individual exposure [23].

### Medical examination

The obligatory medical examination included weight and height measurements, blood sampling, blood pressure, ultrasound measurements (thyroid, abdomen and kidney and adrenals), lung function (starting with a height of 100 cm), and a medical history. Gender and place of residence were also noted. No other information on living conditions or tobacco smoke exposure was collected as a part of this exam. For these obligatory examinations, no parental consent was required.

This work is a first in a series of longitudinal analyses. Here we focus on hematologic function: erythrocytes (red blood cells), leukocytes (white blood cells), and thrombocyte (platelet) count and hemoglobin concentrations. Blood was collected in tubes containing EDTA. A blood count, including erythrocytes, hemoglobin, leukocytes, and thrombocytes, was conducted using Sysmex model F-800 (TOA Medical Electronics Company, Kobe, Japan). Normal blood smears were stained by the standardized azure B-eosin GIEMSA Y Romanowsky procedure. Results of these investigations were entered into a database for the period of 1993 to1998.

### Statistical Analysis

For descriptive purposes, values beyond clinical reference levels were defined in Table 1[24].

**Table 1:** Values beyond clinical reference levels.

Red blood cell count:	younger than 2 years:	≥3.7 × 10^12^cells/L
	2 years and younger than 6:	≥3.9 × 10^12^cells/L
	6 years and younger than 12:	≥4.0 × 10^12^cells/L
	12 years and younger than 18:	≥4.5 × 10^12^cells/L
Hemoglobin:	younger than 6 years:	≥5.59 mmol/L (9 g/dL)
	6 years and younger than 12:	≥7.14 mmol/L (11.5 g/dL
	12 years and older:	≥7.76 mmol/L (12.5 g/dL)
Thrombocytes:	all ages:	≥150 × 10^9^platelets/L
Leukocytes:	1 to 3 years:	≥6–17.5 × 10^9^cells/L
	4 to 7 years:	≥5.5–15.5 × 10^9^cells/L
	8 to 13 years:	≥4.5–13.5 × 10^9^cells/L
	13 years and older:	≥4.5–11.0 × 10^9^cells/L

Data were analyzed using the statistical package SAS Version 9.1 (SAS Institute Inc., Cary, NC, USA). We had six repeated measurements of red and white blood cell markers. To determine whether the measurements agreed over time, we estimated intraclass correlation coefficients for the repeated measurements [25].

To investigate the effect of residential ^137^Cs radiation, we used linear models for repeated measurements (PROC MIXED) [26]. This statistical method compensates for the repeated measurements of each child over the six cross-sectional models (1993–1998). For all markers, we estimated the adjusted means, controlling for confounders.

For the statistical estimation, the regular maximum likelihood method was applied. This model requires that the random effects and the error vector are normally distributed, which was found to be the case for all markers. For the within-subject association, modeling started with an unstructured covariance model which required least constraints. For the repeated measurements, the initial model used serial correlation structures (Gaussian). Based on the Akaike information criterion (AIC), we could simplify the random effect to variance component, but could not simplify the repeated measurement matrix.

The residential measurements of ^137^Cs were ranked into five groups of nearly equal size (PROC RANK), with each rank treated as an indicator variable, facilitating assessing an exposure-response relationship. As for confounding factors, the statistical models included gender, age, and year of measurement. Age was categorized into 18 indicator variables of one-year intervals, with the first group 0–1.5 years old. We used indicator variables for age and year of observation to investigate whether their relationships with the outcome variables were linear. If not, these indicator variables adjusted for non-linearity – for instance as we did with erythrocyte counts and age in girls. To determine whether the change in the distribution of the blood markers over the time of the observation depends also on the residential radiation level, we included an interaction term of time (year of measurement) and ^137^Cs. In addition, we investigated statistical differences in children born before or after the accident.

## Results

Children from 38 different settlements were included in the cohort (Table 2). All villages had different soil measurements of ^137^Cs (Figure 1), ranging from 29 to 879 kBq/m^2 ^(Figure 1). The average measurements shown for each settlement within colored zones are based on contamination modeling. Average values and the colored zones were retrieved from published data [21,22]. Three villages were at the border or outside of the Narodichesky region (Bogdanovka, Gunichu, and Ovruch, total of 3 children). Due to the distribution of ^137^Cs, quintiles of the exposure did not produce equally sized groups (Table 3).

**Table 2:** Villages, soil contamination, number of participating children and measurements ^ψ^

Village	Quintile	Soil contamination ^137^Caesium (kBq/m^2^)	Children		Repeated measurements	
			
			Number	% (n = 1247)	Number	% (n = 4,981)
Brodnik		59	11	0.9	37	0.7

Slavkovci		67	9	0.7	36	0.7
	
Liplanchina		76				

Vyasovka		79	60	4.8	262	5.3

Rubegivka	0	80	50	4.0	205	4.1
	
Radcha		82				

Ovruch		102	4	0.3	14	0.3
	
N. Radcha		104				

Klochki		105	25	2.0	79	1.6

Gresla		102	5	0.4	29	0.6

Norinci		112	71	5.7	305	6.1

Guto-Mariatyn		116	24	1.9	97	1.9

Babinichi		126	9	0.7	45	0.9

Laski		127	75	6.0	317	6.4

Jerev	1	130	28	2.2	111	2.2

Bolotnica		131	53	4.3	182	3.7

Davidki		132	8	0.6	38	0.8

Latashi		134	46	3.7	214	4.3

Snytiche		152	9	0.7	39	0.8
	
Bogdanovka		161				

O. Dorogyn		165	51	4.1	220	4.4

Zakusily		165	35	2.8	120	2.4

N. Dorogin		169	22	1.8	71	1.4

Slavichina		170	7	0.6	30	0.6

Odruby	2	190	6	0.5	11	0.2

Zalesie		198	55	4.4	217	4.4

Suharevka		210	20	1.6	64	1.3

Moteyki		224	29	2.3	132	2.7

Gunichu		225	12	1.0	32	0.6
	
Megeliska		236				

Yajberen		253	19	1.5	90	1.8

Rudnya Basarskaya	3	266	116	9.3	486	9.8
	
Selec		310				

Narodichi		350	279	22.4	1,049	21.1

Basar	4	364	103	8.3	433	8.7

Rossohivske		608	6	0.5	16	0.3
	
Hristinovka		879				

^ψ ^Villages with a small number of children/observations were merged for descriptive purposes.

**Table 3:** Characteristics of the study population

	Children participating (n = 1,247) %	Total number of observations (n = 4,981) %
Sex		
boys	49.1	48.4
Age groups (years) (first participation or year)		
up to 4.5	33.0	15.9
>4.5 to 9.5	37.5	33.7
>9.5 to 14.5	27.4	41.1
>14.5	2.2	9.2
Year of birth		
1979	0.2	0.1
1980	4.6	4.2
1981	4.8	5.3
1982	8.3	9.7
1983	9.7	11.4
1984	9.3	12.0
1985	7.1	9.1
#		
1987	6.7	8.1
1988	8.3	9.0
1989	8.5	7.7
1990	6.8	6.2
1991	6.3	5.3
1992	5.9	5.1
1993	5.0	3.3
1994	5.1	2.3
1995	2.7	1.0
1996	0.8	0.3
Year of first participation (or first entered into the data)		
1993	71.1	17.7
1994	2.4	15.8
1995	2.7	15.8
1996	1.6	15.7
1997	18.9	20.7
1998	3.4	14.3
Quintiles of the area contamination: ^137^Caesium (kBq/m^2^)		
29–112	18.9	19.4
116–156	20.1	20.8
165–253	9.3	19.4
266–310	31.1	9.8
350–879	18.9	30.7

# Children born in 1986 being analyzed and reported in a separate paper

The exact number of children residing in the Narodichesky region between 1993 to 1998 is unknown. Of the 11,400 residents, approximately 2,000 children are children. Data of all exams (1993 to 1998) were included. Officially, participation in the exams was obligatory. However, authorities did not enforce participation. Hence, the term 'obligatory' primarily reflects the opportunity of having annual examinations. Overall, approximately 75% participated in at least one of the examinations (1,459 of about 2,000 children residing in the region). Of these, 1,247 children had a blood sample analyzed (86%), which resulted in 4,981 repeated measurements (Table 3). About one third of the children were 4.5 years and younger, but only 15.9% of the measurements came from these children, indicating a lower participation in blood sampling. The oldest child was born in 1979; the youngest in 1996. Data from the children born in 1986 will be analyzed and reported in a separate paper. Of our cohort, 549 children were born before and 698 after the accident (Table 3). In 75% of the available data, the first participation was documented in 1993, however, the measurements were nearly equally distributed throughout the years. In 1993, we had 886 measurements; 786 in 1994; 787 in 1995; 783 in 1996; 1,030 in 1997; and 709 in 1998.

Because erythrocyte count and hemoglobin concentration measurement represent a nearly identical feature, these markers are highly correlated (r = 0.72, Table 4). The other markers did not show high rank-correlations indicating independent measurements.

**Table 4:** Median, minimal and maximal values, and rank correlation of hematological markers ^#^

Variable	Median	Min	Max	Rank correlation (Spearman) and p-value		
				
				Hemoglobin	Leukocyte	Platelets
Erythrocyte count (10^12^L)	4	2.1	5.7	0.72	0.25	0.22
				0.00	0.00	0.00
Hemoglobin (g/dL)^Φ^	12.3	5.2	16.9		0.18	0.16
					0.00	0.00
Leukocyte count (10^6^L)	6.8	2	18.9			0.35
						0.00
Platelet count (10^9^L)	252	108	670			

# 4,981 repeated measurement of 1,247 children

Φ 1 g/dL equals about 0.6206 mmol/L.

The intraclass-correlation coefficients (ICC) for the four markers over the six years are: erythrocyte count: ICC = 0.4 (5% confidence level: 0.37), for hemoglobin: ICC = 0.59 (5% confidence level: 0.57), for thrombocyte count: ICC = 0.54 (5% confidence level: 0.52), and for leukocyte count: ICC = ICC = 0.53 (5% confidence level: 0.51). These results document substantial stability of the measurements.

Including all cohort children, the repeated measurement analyses (mixed models) showed significant effects for the residential ^137^Cs measurements (quintiles), the time of the medical examination, and the ^137^Cs × time interaction (Table 5). To illustrate the interactions of time and exposure, we extracted mean values for residential exposure classes and years from the mixed models (see details in Additional file 1).

**Table 5:** Statistics of the models including the interaction of area contamination and year of measurement ^#^

	Numerator degree of freedom	Denominator degree of freedom	F-Value	Prob F
Erythrocyte count				
Quintiles of the area contamination with Caesium 137 (kBq/m^2^)	4	3692	12.55	<0.001
Sex	1	3692	12.11	<0.001
Age groups	17	3692	13.42	<0.001
Year of measurement	5	3692	102.42	<0.001
Quintiles of the area contamination: ^137^Caesium (kBq/m^2^) × year	20	3692	5.37	<0.001
				
Hemoglobin				
Quintiles of the area contamination with Caesium 137 (kBq/m^2^)	4	3692	7.28	<0.001
Sex	1	3692	7.72	0.006
Age groups	17	3692	33.43	<0.001
Year of measurement	5	3692	52.68	<0.001
Quintiles of the area contamination: ^137^Caesium (kBq/m^2^) × year	20	3692	4.81	<0.001
				
Platelet count				
Quintiles of the area contamination with Caesium 137 (kBq/m^2^)	4	3690	9.71	<0.001
Sex	1	3690	1.61	0.20
Age groups	17	3690	5.58	<0.001
Year of measurement	5	3690	92.61	<0.001
Quintiles of the area contamination: ^137^Caesium (kBq/m^2^) × year	20	3690	2.08	0.003

# For the illustration of the results see Figure 2 and 3.

**Figure 2:**
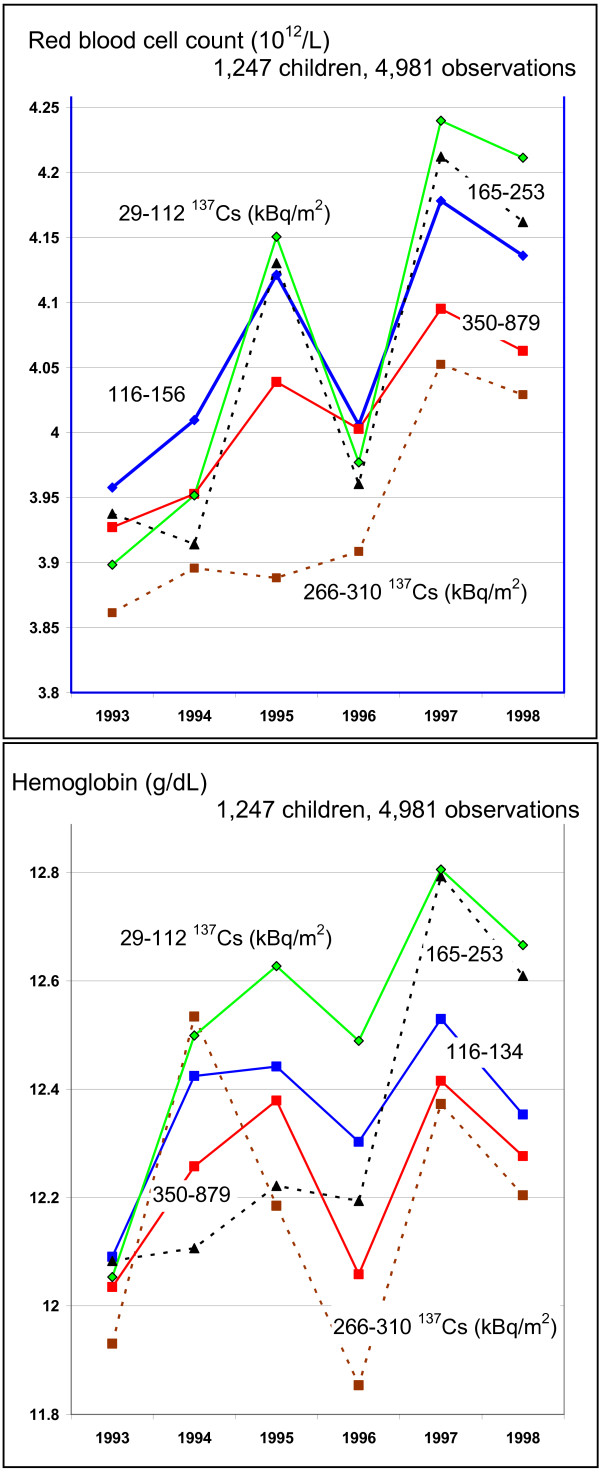
**Changes of erythrocyte counts and hemoglobin over the years of observations by ^137^Cs exposure**. The ^137^Cs contamination is grouped into quintiles. For hemoglobin: 1 g/dL equals about 0.6206 mmol/L.

**Figure 3:**
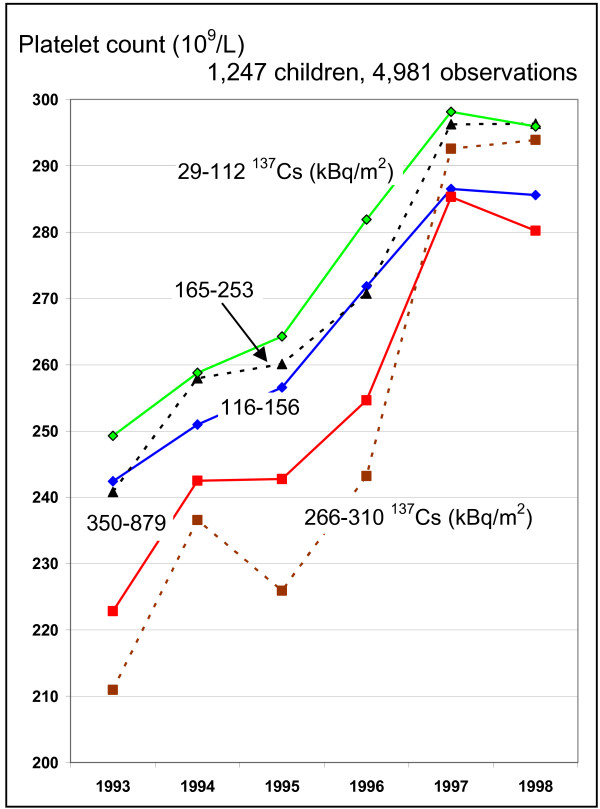
**Changes of platelet counts over the years of observations by ^137^Cs exposure**. The ^137^Cs contamination is grouped into quintiles.

The erythrocyte count shows a minor increase from 1993 to 1998 (Figure 2), starting from below 4 × 10^12 ^cells per liter to more than 4.1 × 10^12 ^cells per liter. The improvement is less in the two groups of children with higher residential exposures; thus the exposure gradient becomes stronger in 1998 compared to 1993. A similar trend is obvious for hemoglobin. Both markers show a dip in 1996, which is not explained by age in general nor by a particular age in girls (age at menarche). For platelet counts, it is obvious that children in all ^137^Cs exposure groups improved over time (Figure 3). The exposure gradient is stronger in 1993, but diminished in 1998. There seems to be no further increase after 1997. Figures 2 and 3 show lower values in 1996 in all exposure groups.

There appears to be exposure-response patterns in all graphs; however, the second to the highest exposure group showed the strongest effect in all four outcomes. Compared to the second highest group, the highest residential exposure group includes a higher proportion of semi-rural children, from Narodichi, the administrative center of the region (Table 2).

For leukocyte counts, the exposure gradient did not change between 1993 and 1998 (Table 6). Starting with a soil contamination of about 165 kBq/m^2^, the leukocyte count is approximately 1 million cells per liter lower in the group of children with the highest soil contamination. A similar reduction was found for children born after the accident in 1986 (data not shown). However, the count increased over the time period from 1993 to 1996.

**Table 6:** Estimate effects of ^137^Caesium and year of measurements on white blood cell counts ^Ψ^

		White blood cell count (10^6^/L) 1,247 children, 4,981 measurement			
Effect		Adj. Mean^#^	Lower	Upper	p

Quintiles of the area contamination: ^137^Caesium (kBq/m^2^)	29–112	6.87	6.58	7.16	Ref.
	116–156	6.88	6.60	7.18	0.94
	165–253	6.40	6.11	6.68	0.01
	266–310	5.95	5.55	6.35	<0.001
	350–879	5.81	5.57	6.04	<0.001

Ψ P-values are based on t-test compared with the reference (Ref.).

# Adjusted for age, sex, born after the accident, and year of measurement

Children born after the accident and thus exposed during pregnancy and childhood showed no ^137^Cs × time interaction (Table 7). For the three outcomes, erythrocyte and platelet count and hemoglobin concentration, the values are significantly lower in the two highest residential exposure groups; the effect is strongest for platelets and white blood cells. Compared with the measurements in 1998 there is an overall improvement over the six years of observation for erythrocyte, platelet, but no clear trend regarding hemoglobin (data not shown).

**Table 7:** Estimated effects of ^137^Caesium and year of measurements on erythrocyte count, hemoglobin, and platelet counts ^Ψ^

		Erythrocyte count (10^12^/L) 698 children, 2,404 measurements				Hemoglobin (g/dL)^Φ ^698 children, 2,404 measurements				Platelet count (10^9^/L) 698 children, 2,403 measurements			
			
Effect		Adj. Mean^#^	5–95% confidence interval		p	Adj. Mean^#^	5–95% confidence interval		p	Adj. Mean^#^	5–95% confidence interval		p
									
Quintiles of the area contamination: ^137^Cs (kBq/m^2^)	29–112	4.02	3.98-	4.06	Ref.	12.2	12.1-	12.3	Ref.	284	274	294	Ref.
	116–156	3.99	3.97-	4.03	0.29	12.0	11.9-	12.2	0.039	273	266	282	0.07
	165–253	4.00	3.93-	3.93	0.42	12.1	12.0-	12.2	0.34	281	273	290	0.52
	266–310	3.88	3.84-	3.93	<0.001	11.8	11.7-	12.0	0.001	255	242	267	<0.001
	350–879	3.96	3.93-	3.99	0.009	12.0	11.0-	12.1	0.033	264	256	271	<0.001

Φ 1 g/dL equals about 0.6206 mmol/L.

Ψ P-value are based on t-test compared with the reference (Ref.) in children born after the accident.

# Adjusted for age, sex, and year of measurement

Fifty-three percent of all erythrocyte counts were below the clinical age-specific reference values, as were 19% of the hemoglobin measurements, 0.7% of the platelet and 26.6% of the white blood cell counts. The proportion of values below the clinical limit decreased from 1993 to 1996. The white blood cell counts also showed improvement, but an increasing proportion of mild leukocytosis (white blood cell count > 11 × 10^9^cells per L; 1993: 0.1%, 1997: 3.2%, 1998: 2.0%).

## Discussion

We analyzed markers of hematopoiesis from 1993 to 1998 and their association with residential radiation exposure and found an adverse effect on erythrocyte, platelet, and white blood cell counts and on hemoglobin concentration. When analyzing the total sample of children irrespective of whether the child was born before or after the accident (exposure occurred in childhood vs. *in utero *and in childhood), we found that the recovery of platelets was more pronounced in children who were exposed to higher residential contamination with ^137^Cs. The recovery of erythrocytes and hemoglobin was smaller in the highly exposed children. White blood cell counts did not show an exposure × time interaction, but remained lower in higher exposed children. Also in children born after the accident there was no such interaction effect. An outcome improvement was obvious in all exposure groups, with the exception of hemoglobin, but the exposure gradient did not diminish over the observation period.

Overall, approximately 75% participated in the examinations (1,459 of about 2,000 children residing in the region); blood samples were available for 63%. Officially, participation in the exams was obligatory. However, authorities did not enforce participation.

This is a dynamic cohort study: children were entering and leaving the study. Regarding non-participation in one exam, lower or higher levels of the three blood count variables in the preceding exam were not associated with missing the next exam. Hence, we have no indication for a selection bias due to differential attrition.

The justification to enter and analyze only data for 1993 to 1998 was due to budgetary limitations. We are seeking funding to extend the data entry and include more repeated measurements. The 1993 to 1998 period was chosen since all exams were established during this timeframe. Another advantage of this period is that it includes a comparable number of children who were born before the accident in 1986 and thereafter. This facilitates a comparison of exposures occurring after birth in children born before the accident in 1986 with exposure in children born after the accident (persistent 137Cs exposure). The variable "born before" addresses two aspects, first, whether the child was exposed to other short-lived radionuclides immediately after the accident (for instance ^131^Iodine, in children born before 1986), and, second, whether the child was exposed to ^137^Cs during pregnancy and in its development (born after).

Originally, we planned to analyze radiation exposure using individual effective equivalent dose in millisievert (mSv), which incorporates total external and internal doses, including direct measurements with whole body counts [27], but there are uncertainties in the time periods in which the measurements were taken and their documentation. In addition, the individual effective equivalent dose was not calculated for every child and exposure models for individual dosimetry were changed over time. Although residential radiation exposure introduces more non-differential misclassification and thus weakens possible associations, those data were our most complete available exposure data set. In addition, the Chernobyl Sasakawa Health and Medical Cooperation project found that individual ^137^Cs levels in the bodies of children were highly correlated (r = 0.7, p < 0.01) with the contamination level in the place of residence [23]. However, compared to the five groups of residential exposure, the individual effective equivalent dose is highest in children in the second to highest residential exposure group (mean individual effective equivalent doses over increasing levels of residential exposure groups: 13.6, 11.7, 16.7, 34.9, 19.6 mSv). Thus, it is likely that the stronger effect in the second highest residential exposure (266–310 ^137^Cs kBq/m^2^) follows from the higher individual dose in this group. We note that the highest exposure group represents children from Narodichi, the largest village of the region (Table 2), whereas the second to highest exposure group includes more children from small villages and rural areas with a larger chance of direct exposure externally and internally from food. Furthermore, compared to the virtue of individual measurements, using residential exposure data has the advantage that it provides the information needed to contribute to improved exposure regulations.

There are other sources of non-differential exposure misclassification, *e.g.*, in landscapes with relatively low levels of radioactive contamination of the soil, the population may receive substantial radiation doses due to their consumption of contaminated food and vice versa. The results also show decreased erythrocyte counts and hemoglobin concentrations in 1996 in all exposure groups (Figures 2 and 3). It is possible that there was an instrument bias in 1996. Another explanation is that the winter in 1996 was colder. Thus, children may have suffered from a reduced supply of vitamins that are essential for the hematopoesis. Since the decline occurred in all exposure groups, this represents a non-differential misclassification. Non-differential misclassifications tend to produce results that underestimate effects and do not present a threat to validity of the findings.

Our data provide only a little information on individual characteristics such as age and gender. One may be inclined to consider that other confounding factors such as parental smoking or diet would increase the internal validity of this study. Against that, we need to understand the setting of this research. The Chernobyl accident affected all groups of the population, irrespective of whether the parent exposed their children to second hand smoke or whether their diet was healthy or not. Thus, smoking and other factors cannot act as confounders, since the accident resulted in a random distribution of radiation in relation to various other health related risk factors. Such a setting is referred to as quasi-experimental or as a randomized natural experiment [28,29]. Although this setting does not require the control of confounders, we cannot exclude other risk factors that may have moderated the adverse effects of radiation (effect modification or interaction). For instance, it is possible that poverty and unhealthy lifestyles have augmented adverse radiation effects. The sample of children was homogeneous. All families were poor villagers with a traditional diet based on local food and comparable domestic conditions. Given the setting of a randomized natural experiment, our results provide compelling evidence for the adverse effects of residential ^137^Cs radiation on hematopoiesis.

Another limitation is that we have only computerized and statistically analyzed data for a limited time-period (1993–1998). However, this is a first step, and there is a clear need to include additional data before 1993 and after 1998. In addition, we had to start with a specific set of health outcomes and chose markers related to hematopoiesis. Other reports that document the various aspects of the Chernobyl accident will follow. These children seem to suffer from multiple diseases and co-morbidities with repeated manifestations, a condition for which the term 'frequently sick child' was defined by Stepanova and coworkers [30].

According to data from the Ukrainian Ministry of Social Protection (January 2005) and Ministry of Emergency, more than half a million children reside in areas with chronic exposure to low radiation due to soil contamination with ^137^Cs. We believe that it is extremely important to analyze not only cancer-related outcomes but also non-neoplastic effects [31], which are much more frequent than cancer. It is surprising that the UN Chernobyl Forum Report [2] did not consider multiple publications by Ukrainian, Russian, and Byelorussian researchers about the excess of non-cancer morbidity in children living in the territory contaminated by the Chernobyl accident [9,32-37]. Because the Chernobyl accident was like few others, there is a gap in knowledge regarding potential health sequelae. The atomic bombing of Japan near the end of World War II did release fission isotopes over a wide area, but the composition of this isotopic contamination was different [38]. The Chernobyl accident involved the release of isotopes built up in the fuel rods over time; these isotopes are far more persistent. As a potential consequence, the decreased blood counts show lasting effects. This was not observed after non-persistent radiation exposure [39]. However, similar effects were reported for the River Techa accident, which happened in 1957 [40]. The exposure was characterized by gamma and beta irradiation due to ^90^Strontium and ^137^Cs. As in children residing in Narodichesky region, adverse hematologic effects were detected. A normalization of the hematologic outcomes occurred only 13 years after the accident [40]. In investigating health sequelae in a cohort of children exposed to the Chernobyl accident, we expect to find associations that have not been reported before. This work is the first in a series using longitudinal epidemiology to uncover long-term effects of the Chernobyl accident on children, leading to a better understanding of how large and persistent the radiation exposure affects the general population.

## Conclusion

More than 10 years after the Chernobyl accident, children in the Narodichesky region, Ukraine, approximately 80 km from Chernobyl, showed decreased counts for red and white blood cells and platelets, and a reduced concentration of hemoglobin associated with persistent residential ^137^Cs exposure. There are compelling reasons to investigate more closely the relationship of radioactive exposure after the Chernobyl accident and health sequelae in children.

## Abbreviations

Intraclass correlation coefficient (ICC), ethylene diamine tetraacetic acid (EDTA)

## Competing interests

The authors declare that they have no competing interests.

## Authors' contributions

Eugenia Stepanova and Vitaliy Vdovenko have contributed to developing the protocol of the medical exams and conducted the examinations and blood analyses. Wilfried Karmaus, Marina Naboka, Vitaliy Vdovenko, Tim Mousseau, John Vena, and Harris Pastides developed the analytical plan. Marina Naboka, Viacheslav M. Shestopalov, and Dwight Underhill provided access and supported the assessment of exposure data. Wilfried Karmaus and Erik Svendsen analyzed the data. All the authors contributed to and approved the final manuscript.

## Supplementary Material

Additional file 1 **Table with confidence limits.** Estimated combined effects of 137Caesium irradiation and year of measurements on erythrocyte count, hemoglobin, and platelet counts and their confidence limits. Click here for file
