# Supplementary material for: Exposure from the Chernobyl accident had adverse effects on erythrocytes, leukocytes, and, platelets in children in the Narodichesky region, Ukraine: A 6-year follow-up study
Source: Environ Health. 2008 May 30;7:21. doi: 10.1186/1476-069X-7-21 (PMC2459146; doi:10.1186/1476-069X-7-21)
Supplement: Additional file 1 — Table with confidence limits. Estimated combined effects of 137Caesium irradiation and year of measurements on erythrocyte count, hemoglobin, and platelet counts and their confidence limits. [file 1476-069X-7-21-S1.doc]

Additional file: table with confidence limits.doc, 90 k

Estimated combined effects of ^137^Caesium irradiation and year of measurements on erythrocyte count, hemoglobin, and platelet counts and their confidence limits

| Year of medical exam | Quintiles of the area contami-tion: ^137^Cs (kBq/m^2^) | Erythrocyte count (10^12^/L) | | | Hemoglobin (g/dL)^^ | | | Platelet count (10^9^/L) | | |
| --- | --- | --- | --- | --- | --- | --- | --- | --- | --- | --- |
| time |  | Adjusted Mean^#^ | 5-95% confidence interval | | Adjusted Mean^#^ | 5-95% confidence interval | | Adjusted Mean^#^ | 5-95% confidence interval | |
| 1993 | 29-112 | 3.90 | 3.86 | 3.94 | 12.1 | 11.9 | 12.2 | 249.3 | 239.7 | 258.9 |
| 1993 | 116-156 | 3.96 | 3.92 | 4.00 | 12.1 | 11.9 | 12.2 | 242.4 | 232.7 | 252.1 |
| 1993 | 165-253 | 3.94 | 3.90 | 3.98 | 12.1 | 11.9 | 12.2 | 240.8 | 231.2 | 250.5 |
| 1993 | 266-310 | 3.86 | 3.80 | 3.92 | 11.9 | 11.7 | 12.1 | 211.0 | 197.2 | 224.8 |
| 1993 | 350-879 | 3.93 | 3.89 | 3.96 | 12.0 | 11.9 | 12.2 | 222.8 | 214.6 | 231.0 |
| 1994 | 29-112 | 3.95 | 3.91 | 4.00 | 12.5 | 12.4 | 12.6 | 258.8 | 248.8 | 268.8 |
| 1994 | 116-156 | 4.01 | 3.97 | 4.05 | 12.4 | 12.3 | 12.6 | 251.0 | 241.1 | 260.9 |
| 1994 | 165-253 | 3.91 | 3.87 | 3.96 | 12.1 | 12.0 | 12.3 | 257.9 | 247.8 | 268.0 |
| 1994 | 266-310 | 3.90 | 3.84 | 3.96 | 12.5 | 12.3 | 12.7 | 236.6 | 223.0 | 250.1 |
| 1994 | 350-879 | 3.95 | 3.92 | 3.99 | 12.3 | 12.1 | 12.4 | 242.5 | 234.2 | 250.7 |
| 1995 | 29-112 | 4.15 | 4.11 | 4.20 | 12.6 | 12.5 | 12.8 | 264.3 | 254.2 | 274.4 |
| 1995 | 116-156 | 4.12 | 4.08 | 4.16 | 12.4 | 12.3 | 12.6 | 256.6 | 246.8 | 266.3 |
| 1995 | 165-253 | 4.13 | 4.09 | 4.17 | 12.2 | 12.1 | 12.4 | 260.1 | 250.0 | 270.2 |
| 1995 | 266-310 | 3.89 | 3.83 | 3.95 | 12.2 | 12.0 | 12.4 | 225.9 | 212.1 | 239.7 |
| 1995 | 350-879 | 4.04 | 4.00 | 4.08 | 12.4 | 12.3 | 12.5 | 242.7 | 234.4 | 251.0 |
| 1996 | 29-112 | 3.98 | 3.93 | 4.02 | 12.5 | 12.3 | 12.6 | 281.9 | 271.8 | 291.9 |
| 1996 | 116-156 | 4.01 | 3.96 | 4.05 | 12.3 | 12.2 | 12.4 | 271.8 | 262.3 | 281.3 |
| 1996 | 165-253 | 3.96 | 3.92 | 4.00 | 12.2 | 12.1 | 12.3 | 270.7 | 261.0 | 280.5 |
| 1996 | 266-310 | 3.91 | 3.85 | 3.97 | 11.9 | 11.7 | 12.1 | 243.2 | 229.4 | 257.0 |
| 1996 | 350-879 | 4.00 | 3.97 | 4.04 | 12.1 | 11.9 | 12.2 | 254.6 | 246.6 | 262.7 |
| 1997 | 29-112 | 4.24 | 4.20 | 4.28 | 12.8 | 12.7 | 12.9 | 298.1 | 289.1 | 307.2 |
| 1997 | 116-156 | 4.18 | 4.14 | 4.22 | 12.5 | 12.4 | 12.7 | 286.5 | 277.7 | 295.3 |
| 1997 | 165-253 | 4.21 | 4.17 | 4.25 | 12.8 | 12.7 | 12.9 | 296.3 | 287.6 | 305.0 |
| 1997 | 266-310 | 4.05 | 4.00 | 4.11 | 12.4 | 12.2 | 12.6 | 292.6 | 279.9 | 305.3 |
| 1997 | 350-879 | 4.10 | 4.06 | 4.13 | 12.4 | 12.3 | 12.5 | 285.3 | 278.2 | 292.4 |
| 1998 | 29-112 | 4.21 | 4.16 | 4.26 | 12.7 | 12.5 | 12.8 | 295.9 | 285.5 | 306.4 |
| 1998 | 116-156 | 4.14 | 4.09 | 4.18 | 12.4 | 12.2 | 12.5 | 285.6 | 275.8 | 295.4 |
| 1998 | 165-253 | 4.16 | 4.12 | 4.21 | 12.6 | 12.5 | 12.8 | 296.4 | 286.4 | 306.3 |
| 1998 | 266-310 | 4.03 | 3.96 | 4.09 | 12.2 | 12.0 | 12.4 | 293.9 | 279.6 | 308.2 |
| 1998 | 350-879 | 4.06 | 4.02 | 4.10 | 12.3 | 12.2 | 12.4 | 280.2 | 271.9 | 288.5 |

 1 g/dL equals about 0.6206 mmol/L.

# Adjusted for age and sex.
